# Revisiting the Effect of Slag in Reducing Heat of Hydration in Concrete in Comparison to Other Supplementary Cementitious Materials

**DOI:** 10.3390/ma11101847

**Published:** 2018-09-27

**Authors:** Hoon Moon, Sivakumar Ramanathan, Prannoy Suraneni, Chang-Seon Shon, Chang-Joon Lee, Chul-Woo Chung

**Affiliations:** 1 Department of Architectural Engineering, Pukyong National University, Yongso-ro 45, Nam-gu, Busan 48513, Korea; mhmoona@naver.com; 2 Department of Civil, Architectural and Environmental Engineering, University of Miami, Coral Gables, FL 33146, USA; sxr1063@miami.edu (S.R.); suranenip@miami.edu (P.S.); 3 Department of Civil and Environmental Engineering, Nazarbayev University, Astana 010000, Kazakhstan; chang.shon@nu.edu.kz; 4 Department of Architectural Engineering, Chungbuk National University, Chungdae-ro 1, Seowon-gu, Cheong-ju 28644, Korea; cjlee@chungbuk.ac.kr

**Keywords:** blast furnace slag, calcium sulfate, heat of hydration, maximum temperature rise, adiabatic calorimeter, semi-adiabatic calorimeter, isothermal calorimeter

## Abstract

Blast furnace slag (SL) is an amorphous calcium aluminosilicate material that exhibits both pozzolanic and latent hydraulic activities. It has been successfully used to reduce the heat of hydration in mass concrete. However, SL currently available in the market generally experiences pre-treatment to increase its reactivity to be closer to that of portland cement. Therefore, using such pre-treated SL may not be applicable for reducing the heat of hydration in mass concrete. In this work, the adiabatic and semi-adiabatic temperature rise of concretes with 20% and 40% SL (mass replacement of cement) containing calcium sulfate were investigated. Isothermal calorimetry and thermal analysis (TGA) were used to study the hydration kinetics of cement paste at 23 and 50 °C. Results were compared with those with control cement and 20% replacements of silica fume, fly ash, and metakaolin. Results obtained from adiabatic calorimetry and isothermal calorimetry testing showed that the concrete with SL had somewhat higher maximum temperature rise and heat release compared to other materials, regardless of SL replacement levels. However, there was a delay in time to reach maximum temperature with increasing SL replacement level. At 50 °C, a significant acceleration was observed for SL, which is more likely related to the pozzolanic reaction than the hydraulic reaction. Semi-adiabatic calorimetry did not show a greater temperature rise for the SL compared to other materials; the differences in results between semi-adiabatic and adiabatic calorimetry are important and should be noted. Based on these results, it is concluded that the use of blast furnace slag should be carefully considered if used for mass concrete applications.

## 1. Introduction

Controlling the temperature rise in concrete structures employing large volumes of concrete (mass concrete) is critical to prevent thermal cracking caused by large temperature differentials between the concrete surface and its interior even without external structural restraint. Large concrete structures like high-rise buildings and hydro-electric power plants contain structural components and foundations with massive concrete sections. The heat of hydration either dissipates from these concrete sections slowly or is not dissipated locally due to the greater thickness of concrete and its low thermal conductivity [1]. This leads to large temperature differentials between the concrete surface and its interior as well as a self-insulation effect and consequently may result in cracking [2,3]. Concrete exposed to high thermal stresses can suffer from loss of structural integrity and poor durability due to microcracking associated with thermal stresses [4].

There are several ways to control the heat of hydration of concrete. These include cooling the mixing water, cooling the aggregate, using ASTM Type II moderate heat cement or Type IV low-heat cement, keeping cement contents to a minimum level, using supplementary cementitious materials (SCMs), and choosing proper curing methods [5]. Among these methods, it is common to use cement with partial replacement of SCMs because the early-age heat of hydration generated by the combined use of cement and SCMs is generally smaller than that produced by Portland cement alone [6,7]. SCMs that have been used for such purposes include blast furnace slag (SL), fly ash (FA), silica fume (SF), and metakaolin (MK) [8,9,10,11,12]. Materials such as SF and MK reduce the overall temperature rise of the concrete depending on temperature conditions, but the effect is lesser due to their higher reactivity and lower replacement levels [13].

Traditionally, SL, which is an amorphous calcium aluminosilicate material, has been considered to exhibit both pozzolanic and latent hydraulic activity [14]. Reactivity of SL is controlled by chemical composition, glass content, fineness, and particle size distribution [14,15]. Pure SL reacts with water at a very low rate or may not react at all [16,17]. Its reactivity is, however, increased as pH increases [18,19]. Because the reactivity of SL can also be increased by the presence of calcium sulfates [14,20,21], commercial SL typically contains some type of calcium sulfate [22,23]. Increasing SL fineness by grinding may also be performed to enhance the reactivity of SL. Therefore, it is expected that such SLs would be less effective to control the heat of hydration of a large volume of concrete.

It is generally accepted that the use of SL decreases the maximum temperature rise of the concrete. Previous studies have shown that total heat of hydration in isothermal conditions decreases as the SL replacement level increases [24,25]. The degree of hydration of cement and the degree of reaction of SL in cement blended with SL increase with increasing curing temperature and decrease with increasing SL replacement level [26,27]. However, these results were obtained by using traditional types of SL and not by using modified, blended, or pre-treated slag.

Moreover, the measurements of the heat of hydration in previous research were mostly conducted using either cumulative heat release data under ambient isothermal conditions or semi-adiabatic testing, and not using true adiabatic calorimetry. Tänzer et al. [28] and Özbay et al. [4] have quantified the temperature rise of concrete using the cumulative heat of hydration obtained from isothermal conditions (around 23–27 °C). Ng et al. [29] found that the measurement of temperature rise in semi-adiabatic conditions resulted in substantial heat loss during measurements. Semi-adiabatic temperature rise combined with numerical analysis has also been frequently used to estimate the maximum temperature rise of the mass concrete [30,31,32]. However, these methods disregard the effect of the increased temperature on the hydration kinetics. Since SL has latent hydraulic characteristics [21,33,34,35], increasing temperature may result in a significant acceleration.

A very limited number of studies have utilized true adiabatic testing setups. De Schutter [36] utilized both isothermal and adiabatic calorimetry to study reaction kinetics of cement paste including SL. Gruyaert et al. [37] further developed this approach and investigated the hydration kinetics of cement paste with SL and showed that the maximum heat release of the cement paste with SL can be similar to that of plain cement paste in certain conditions. However, their work focused on quantifying the degree of hydration, rather than demonstrating the effect of SL on the maximum adiabatic temperature rise of the concrete. In addition, they used a relatively higher water-to-binder ratio (w/b = 0.5), which is a suitable choice for normal hydration studies but does not represent the behavior of high-performance concrete containing SL which has typical w/b values below 0.40. Since available data on the effect of SL on the temperature rise of mass concrete contains some drawbacks, it is necessary to measure the heat of hydration of the concrete containing SL under a true adiabatic condition and compare the results with those from other SCMs.

The purpose of this work is to determine whether SL is an effective material for temperature control of a large volume of concrete with lower w/b. For this purpose, the adiabatic temperature rise of concrete containing various SCMs was measured, and compared with the data from semi-adiabatic temperature rise. Isothermal calorimetry analyses were performed at two different temperature conditions (23 °C and 50 °C) in order to investigate the effect of temperature on reaction kinetics of the SCMs in cement paste. The 28-day compressive strength of each concrete cured in 23 °C lime saturated solution was also determined.

## 2. Materials and Experimental Procedures

### 2.1. Materials Characterization

Cementitious materials used in this study include ASTM Type I ordinary Portland cement (OPC) manufactured by Ssangyong Cement Industrial Co., Ltd. (Seoul, Korea), Grade 80 Blast furnace slag (typically lower strength activity index and fineness than Grades 100 and 120), ASTM Class F fly ash, undensified silica fume, and metakaolin. Chemical compositions of all materials were analyzed using Shimadzu X-ray Fluorescence spectroscopy XRF-1700 (Kyoto, Japan). The densities of the materials were measured by Micromeritics AccuPyc II 1340 pycnometer (Norcross, GA, USA). Table 1 shows the chemical compositions and densities of the materials used in this study.

**Figure 1:**
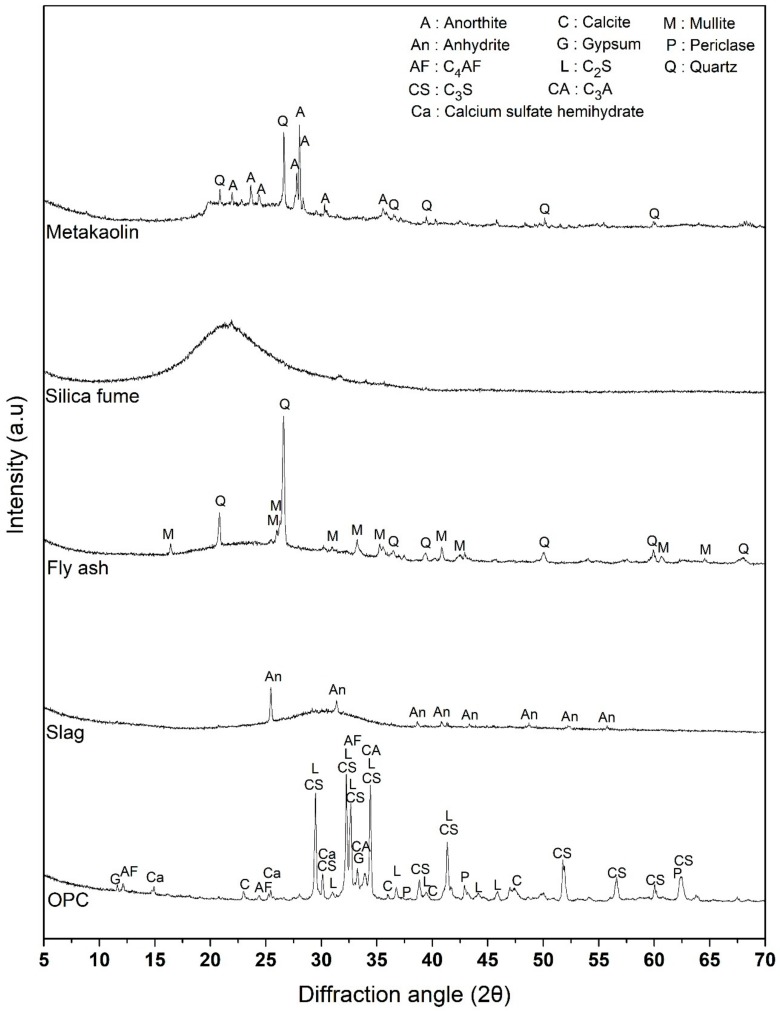
XRD patterns of cementitious materials used for the experiments.

Mineralogical analyses of all materials were also performed using Rigaku Ultima IV X-ray diffractometer (Tokyo, Japan) with Cu-Kα radiation. Working voltage and current were set to 40 kV and 40 mA, respectively. The scanning 2θ angle was varied from 5° to 70°. The results are shown in Figure 1. Four main phases in OPC, tricalcium silicate (C_3_S), dicalcium silicate (C_2_S), tricalcium aluminate (C_3_A), and tetracalcium aluminoferrite (C_4_AF), were identified in the diffraction pattern. Two types of calcium sulfate polymorphs, gypsum and hemihydrate, and periclase were also observed. Blast furnace slag consists of amorphous calcium aluminosilicate and anhydrite. Fly ash contains quartz and mullite and an amorphous band at lower diffraction angles, which is a typical characteristic of low calcium fly ash [38]. Silica fume consists of amorphous silica. Metakaolin contains calcium-rich anorthite and quartz, and large amorphous humps which likely represent low calcium aluminosilicates similar to that of fly ash were observed between 20 and 30°.

### 2.2. Mixture Proportions and Mixing Procedure

Mixture proportions of the concrete used for this work are shown in Table 2. The water to binder ratio (w/b) of the concrete was 0.35. The proportion of fine aggregate within the total aggregate amount (s/a) was 40%. The source of the fine and coarse aggregate was crushed stone. The maximum size of the coarse aggregate was 20 mm.

While the replacement level of SF, MK, and FA was fixed at 20%, two replacement levels (20 and 40%) of SL were investigated. A polycarboxylate-based water reducing admixture (0.2 liquid wt.% of cement) was used to maintain proper workability of concrete (slump value of 150 mm). The polycarboxylate admixture was added along with the mixing water. Before mixing, all materials were preconditioned in a laboratory with constant temperature (23 ± 0.5 °C) for a day to minimize temperature differences of each material.

For mixing procedure, crushed sand and stone were dry-mixed for 2 min. Cement and SCM were added and dry-mixed for additional 3 min. Water was then added to the dry-mixture, and continuously wet-mixed for 5 min. After mixing, concrete was poured into the containers for adiabatic and semi-adiabatic calorimeter as well as for 10 cm × 20 cm cylindrical molds for the 28-day compressive strength test. Casting of concrete were done using placements of 3 equal layers with 25 times rodding on each layer, in addition to some vibrations using a rubber hammer.

### 2.3. Adiabatic Temperature Rise

**Figure 2:**
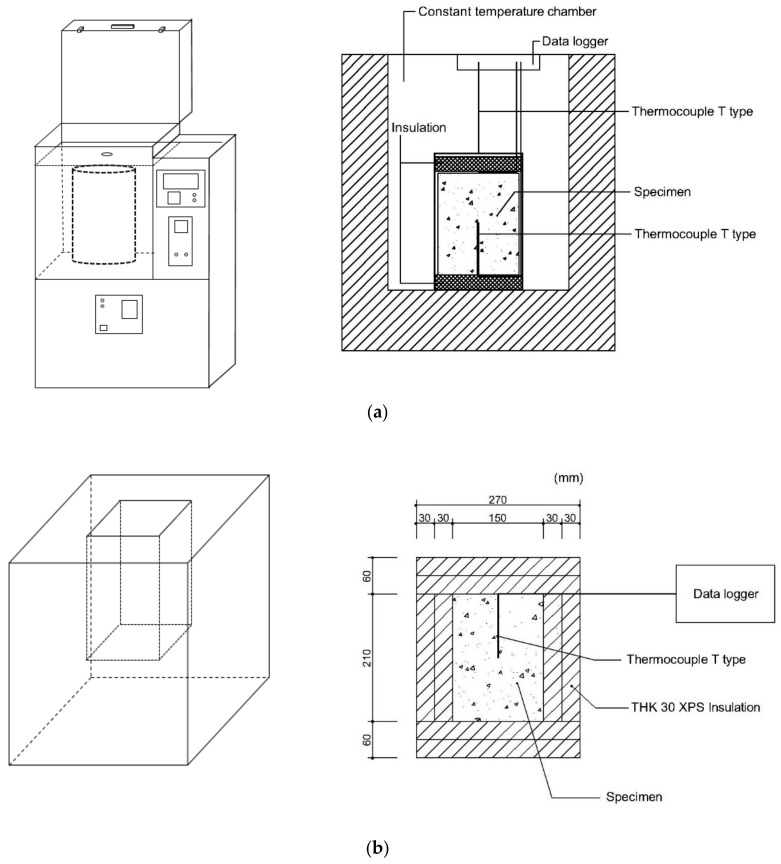
Schematic diagrams of (**a**) adiabatic and (**b**) semi-adiabatic calorimeter.

The temperature rise of the concrete was measured using an adiabatic calorimeter, Tokyo Riko ACM-120HA (Gunma, Japan). The machine was designed to compensate the heat loss during the measurement, which was achieved by controlling the temperature of the specimen container to be the same as the temperature at the center of the specimen. The schematic diagram for the adiabatic calorimeter setup is shown in Figure 2a.

Prior to the experiment, the adiabatic calorimeter was stabilized at 20 °C. Four liters of freshly mixed concrete were poured into the container, which was located inside the thermostat. A type T thermocouple was located at the center of the concrete specimen. The temperature rise was measured every 5 min for first the 24 h after the start of measurement and then subsequently was measured every 10 min. The ambient temperature of the laboratory was maintained at 23 ± 0.5 °C.

### 2.4. Semi-Adiabatic Temperature Rise

A semi-adiabatic temperature rise measurement was performed for comparison with results from the adiabatic temperature measurement. A custom-made experimental set-up was used. Figure 2b shows a schematic diagram of the semi-adiabatic container. The semi-adiabatic container was made of 60 mm thick expanded polystyrene using plastic bond and heat insulating duct tape. The same amount of concrete (4 L) used in adiabatic temperature rise test was used for measurement of semi-adiabatic temperature rise. Type T thermocouple was located at the center of the specimen, which was placed in the semi-adiabatic container. The temperature rise was measured using a Pico Technology TC-08 thermocouple data logger (Cambridgeshire, UK). The measurement was done every minute. The temperature of the laboratory for measuring the hydration temperature was maintained at 23 ± 0.5 °C. 

### 2.5. Isothermal Calorimetry

Isothermal calorimetry was used to determine the effect of the SCMs on the hydration of the cement paste. In order to isolate the effect of the SCMs, testing was conducted with cement paste samples similar to Table 2, but water reducing admixture was not used. For the mixing, 40 g of pastes were hand mixed for four minutes using a spatula. Hand mixing was adapted due to small sample sizes needed for the isothermal calorimetry test. After mixing, approximately 6–7 g of sample was placed in a glass ampoule and lowered into the isothermal calorimeter (TAM Air, TA Instruments, New Castle, DE, USA) and heat release data was collected for 7 days. Isothermal calorimetry was carried out at two different temperatures: 23 ± 0.01 °C and 50 ± 0.05 °C, in order to study the effect of temperature on the hydration kinetics.

### 2.6. Thermal Analysis

This was performed using samples from isothermal calorimetry measurement using a thermogravimetric analyzer (TGA 55, TA Instruments, New Castle, DE, USA). After 7 days of isothermal calorimetry measurement, samples were taken out of the ampoule without stopping the hydration and then ground using mortar and pestle. Approximately 30–40 mg of the sample was placed in the crucible and tested. The temperature was ramped at the rate of 10 °C/min to 1000 °C to quantify the amount of calcium hydroxide that is present in the cement paste mixtures. The calcium hydroxide content was determined based on its mass loss from 380 °C to 460 °C by using the modified tangential method in which the mass loss is evaluated by using the vertical distance between two tangential lines drawn to the mass loss curve [39]. To minimize the experimental error, all the TGA experiments were finished within 12 h after termination of isothermal calorimetry measurement. 

### 2.7. Compressive Strength

The 28-day compressive strength of concrete was measured to quantify the effect of SCMs on mechanical properties. After mixing, fresh concrete was poured into 100 mm × 200 mm cylindrical mold to fill 1/3 of its volume. The concrete was rodded 25 times and the mold was vibrated using a rubber hammer. This procedure was repeated until the cylindrical mold was completely filled with concrete. The top of the container was covered with plastic wrap and stored for 24 h. The concrete was demolded after 24-h and immersed in a saturated calcium hydroxide solution at 23 ± 0.5 °C for 27 days. Compressive strength measurement followed ASTM C 39, maintaining the loading rate of 0.2 MPa/s.

## 3. Experimental Results

### 3.1. Adiabatic Temperature Rise

**Figure 3:**
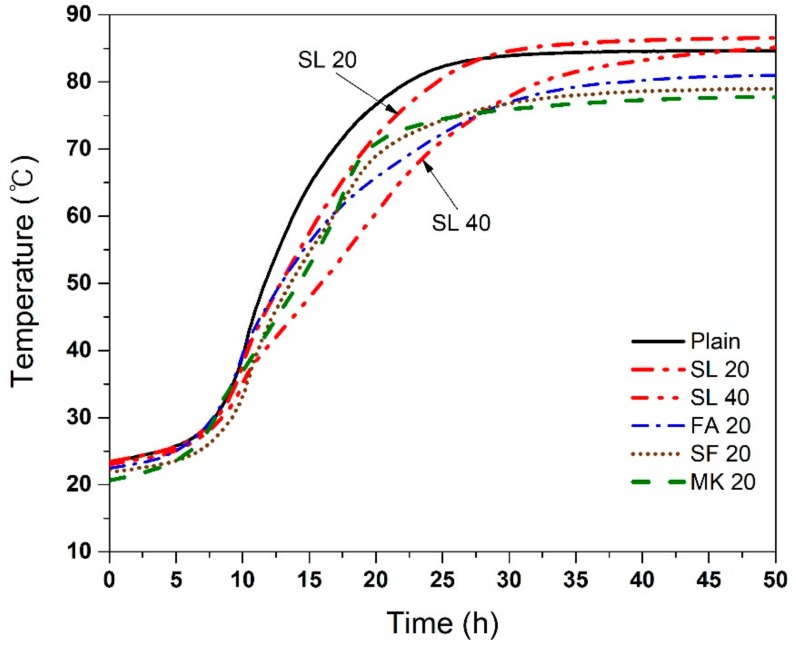
Adiabatic temperature rise of concrete with various SCMs. The difference in the maximum temperature of repeat specimens was generally <3%.

The adiabatic temperature rise of the concrete with various SCMs is presented in Figure 3. Plain concrete without SCMs showed the fastest temperature rise. It reached a maximum temperature of 84.62 °C at 41.83 h. The use of 20% SF, MK, and FA not only reduced the maximum temperature but also delayed the time to reach the maximum temperature. The maximum temperatures and times to reach the maximum temperature are summarized in Table 3. The use of SL did not decrease the maximum temperature of the concrete and even showed slightly higher maximum temperature than that of plain concrete. The effect of SL replacement in adiabatic temperature rise was only to delay the time to reach the maximum temperature. As shown in Table 3, the maximum temperatures were 86.69 °C at 57.83 h with 20% SL and 86.98 °C at 90.67 h with 40% SL, respectively. The mixtures, ordered in ascending order of maximum temperature, are MK 20 < SF 20 < FA 20 < Plain < SL 20 < SL 40. These results suggest that SL may not reduce the maximum temperature of the concrete and is contrary to expected results [7,25,40]. Further analysis is presented in the Discussion Section.

### 3.2. Semi-Adiabatic Temperature Rise

**Figure 4:**
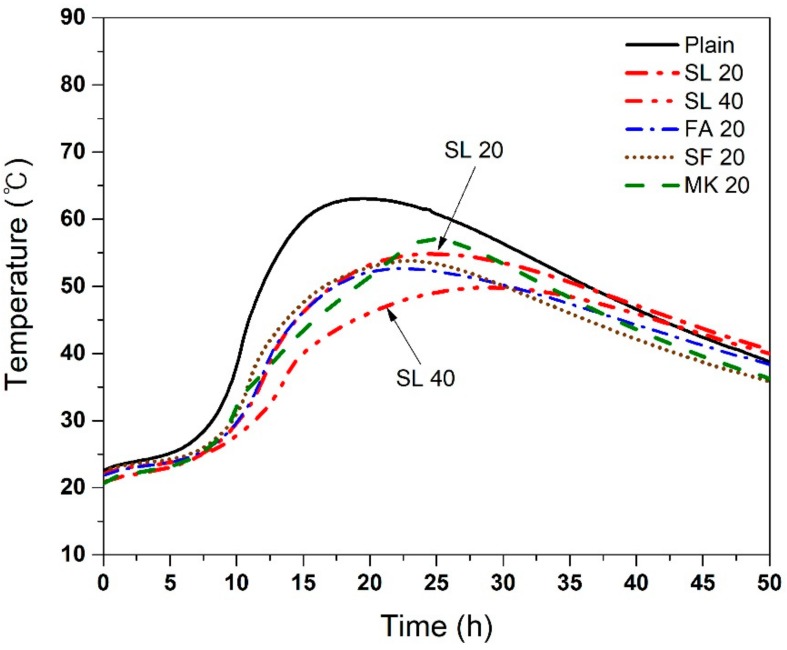
Semi-adiabatic temperature rise of the concrete with various SCMs. The difference in the maximum temperature of repeat specimens was generally <5% when tested in laboratory with constant temperature.

Figure 4 shows a semi-adiabatic temperature rise of the concrete with various SCMs. The maximum temperatures and times to reach the maximum temperature are also summarized in Table 3. The use of all SCMs reduced the maximum temperature of the concrete. The mixtures, ordered in ascending order of maximum temperature, are SL 40 < FA 20 < SF 20 < SL 20 < MK 20 < Plain. It is critical to note that the SL did not show the same results as presented in adiabatic conditions. In semi-adiabatic testing condition, the increase in the replacement level of SL reduced the maximum temperature rise of the concrete, similar to FA. The results from the semi-adiabatic testing condition are in line with results from literature [7,25,40]. It should be noted that when the specimen is exposed to semi-adiabatic conditions, the concrete with the SL shows lower maximum temperature than plain concrete regardless of the amount of SL. These differences are likely due to the continuous heat losses during semi-adiabatic measurements. It is possible that the heat losses have a substantial effect on the maximum temperature reached, especially with SL. This suggests that SL can be used to reduce heat of hydration of concrete in most cases when heat losses from the structure are expected.

### 3.3. Isothermal Calorimetry

**Figure 5:**
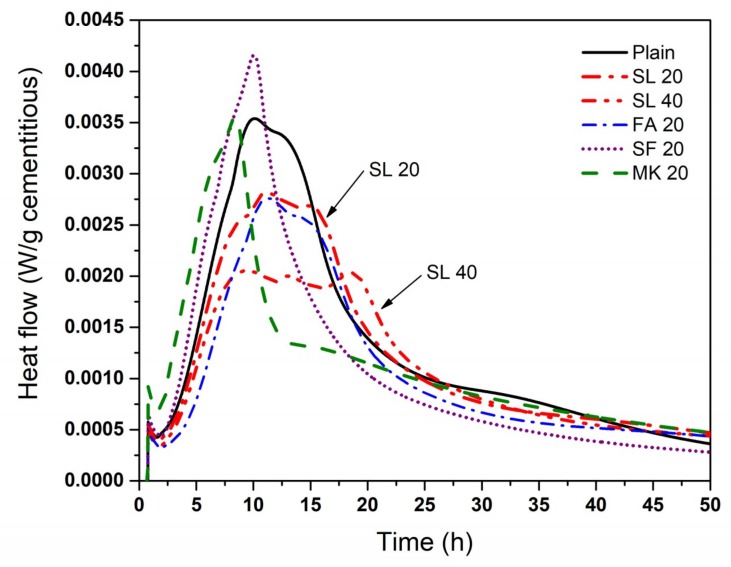
Heat flow of cement paste with various SCMs at 23 °C.

Isothermal calorimetry was performed to investigate the effect of temperature on the reaction kinetics. Figure 5 shows the heat flow of cementitious pastes with various SCMs at 23 °C. The mixtures, ordered in ascending order of reaction kinetics (earliest peak time), are SL 40 < FA 20 < SL 20 < Plain < SF 20 < MK 20, though exact peak time is difficult to determine with the slags due to the presence of multiple peaks. The order of peak magnitudes was the highest for SF 20, followed by MK 20 and plain cement paste that showed similar peak magnitude. Such results may be explained by the filler effect: incorporation of finely powdered SCMs such as MK and SF into cement accelerates the early hydration of cement by providing additional surface area for nucleation and growth of hydrates [11,41,42]. The use of FA and SL results in a delay in the hydration kinetics and a reduction in the maximum peak height.

**Figure 6:**
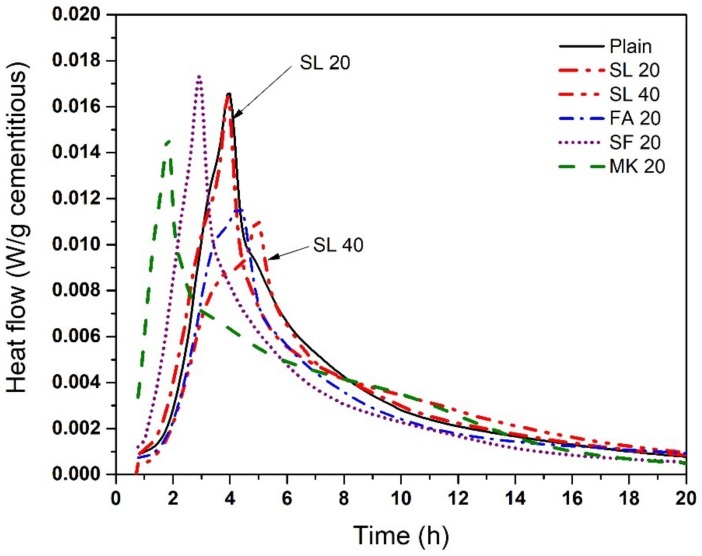
Heat flow of cement paste with various SCMs at 50 °C.

Isothermal calorimetry results of cementitious paste with various SCMs at 50 °C are shown in Figure 6. The mixtures, ordered in ascending order of reaction kinetics (earliest peak time), are SL 40 < FA 20 < SL 20 = Plain < SF 20 < MK 20; and the order for peak magnitude is the same with the exception that MK 20 showed a lower peak magnitude than SF 20 SL 20, and cement paste. Peak heights at 50 °C occur approximately 6 h earlier than at 23 °C, however, general trends at both temperatures were similar.

**Figure 7:**
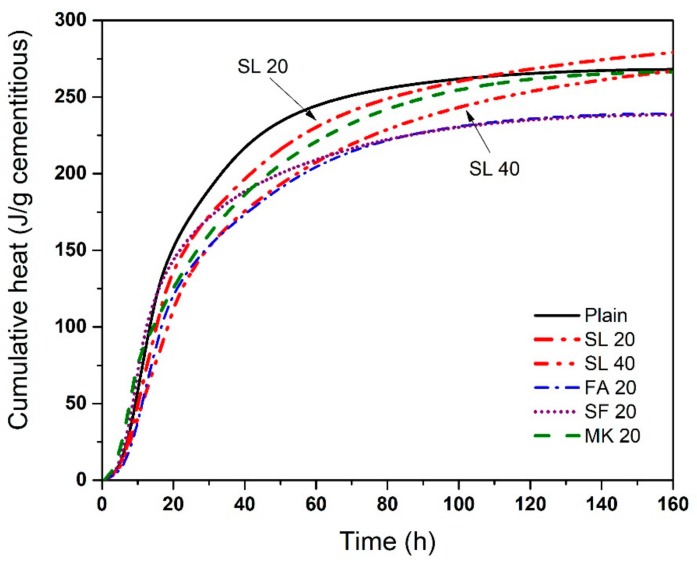
Cumulative heat release of cement paste with various SCMs at 23 °C. The difference in the cumulative heat of repeat specimens was generally < 3%.

Figure 7 shows the cumulative heat release of cementitious paste with various SCMs at 23 °C. Initial cumulative heat evolution is highest for plain cement paste (until about 105 h) but at later ages, SL 20 showed the highest cumulative heat evolution. SL 40 showed slower cumulative heat evaluation than that of plain cement paste, but it eventually exceeds the heat evolution after 160 h. The mixtures, ordered in ascending order of ultimate cumulative heat, are SF 20 < FA 20 < MK 20 < Plain < SL 40 < SL 20. While the exact trend is not the same as the adiabatic temperature rise trend, the authors note that in both cases, FA 20, MK 20, and SF 20 show values below the plain mixture, while the SL 20 and SL 40 mixtures show values greater than the plain mixture. 

**Figure 8:**
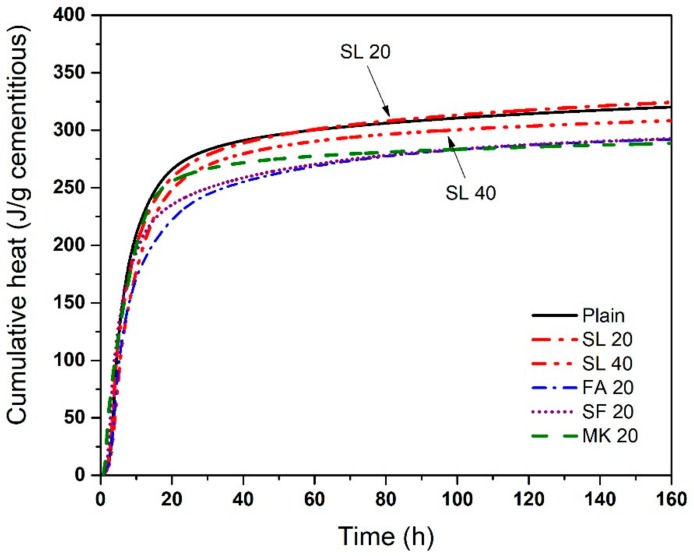
Cumulative heat release of cement paste with various SCMs at 50 °C. The difference in the cumulative heat of repeat specimens was generally <3%.

Cumulative heat release of cementitious pastes with various SCMs at 50 °C is shown in Figure 8. The mixtures, ordered in ascending order of ultimate cumulative heat, are MK 20 < FA 20 < SF 20 < SL 40 < Plain < SL 20. Initial cumulative heat evolution is highest for plain cement paste, but at later ages, it is higher in cement paste with 20% SL, similar to results at 23 °C. Other SCMs show a slower cumulative heat evolution. Cement paste with 20% SF and 20% FA showed similar cumulative heat release although cement paste with SF showed a faster reaction. The orders of cumulative heat release at 50 °C and 23 °C are somewhat different, but in both cases, the highest values of the heat release among the SCMs is seen in mixtures with SL. Elevated temperature accelerates reaction of the cement and the SCMs—cumulative heat release values at 50 °C are higher than values at 23 °C by approximately 18% (17% on average for mixtures with SCMs and 19% for the plain mixture). As SL shows highest temperature and heat release, compared to the other SCMs and the plain mixture, it is suggested that SL containing anhydrite used for this work may potentially be activated and have an accelerated reaction at normal and higher temperatures.

As these pastes did not use a superplasticizer, a full dispersion of the cement and SCMs is likely not achieved, which may explain the somewhat lower cumulative heat release of the pastes with SF and MK. However, it should be noted that these materials also showed lower maximum temperature in the adiabatic test. In addition, the filler effect, which increases cement degree of hydration in the presence of SCMs [43,44], may not occur in a uniform manner with all SCMs. Specifically, it could be possible that the SL may accelerate the cement hydration to a greater extent than the other SCMs. It is also known that aluminate phases are accelerated more than silicate phases at higher temperatures [26,45], and it is possible that certain SCMs, such as SL, further accelerate the aluminate reaction. While this suggests that the effects of the SL are more complex than a simple acceleration in its reactivity, it does not change the conclusion that such an SL is potentially unsuitable for mass concrete structural applications.

### 3.4. Thermal Analysis

**Figure 9:**
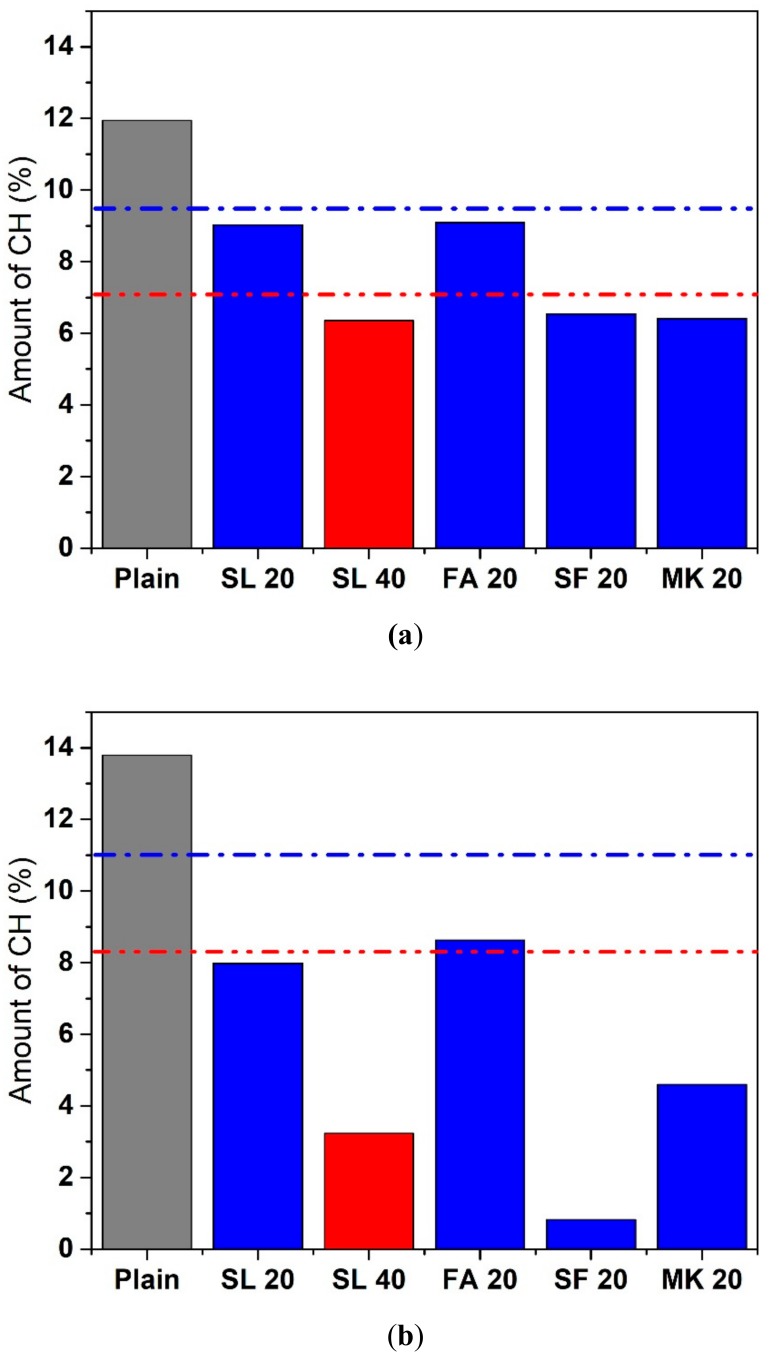
Amount of calcium hydroxide in cement pastes after 7 days (**a**) 23 °C and (**b**) 50 °C (the blue and red dash-dot lines indicate 80% and 60% of the calcium hydroxide amounts in the plain cement paste, respectively. The difference in the calcium hydroxide amounts determined from repeat specimens was generally <3%.

Figure 9a shows the amount of calcium hydroxide (CH) in the cement paste with various SCMs at 23 °C after 7 days (units of % paste mass). The mixtures, ordered in ascending order of CH contents, are MK 20 < SL 40 < SF 20 < FA 20 < SL 20 < Plain. This trend is unsurprisingly different from heat and temperature trends as slag is more (latent) hydraulic than pozzolanic. At 23 °C, plain cement paste had 11.94% CH. The replacement of all SCMs reduced the amount of CH. The amount of CH in the cement paste with 20% SF and MK was 6.54% and 6.41%, respectively. The amount of CH in cement paste with 20% FA was 9.09%. With 20% and 40% replacement of SL, the amount of CH was 9.02% and 6.35%. Supposing that 20% and 40% replacement of SL would reduce the amount of CH by its replacement level (neglecting the filler effect), the amounts of CH that were supposed to be produced with 20% and 40% reduction in portland cement would be 9.55% and 7.16%, respectively. The amounts of CH in cement paste with 20% and 40% replacement of SL (9.02% and 6.35%) were smaller than those values, thereby indicating some degree of pozzolanic reaction has occurred due to the SL at 23 °C.

Figure 9b shows the amount of CH in the cementitious paste with various SCMs at 50 °C after 7 days. The mixtures, ordered in ascending order of CH contents, are SF 20 < SL 40 < MK 20 < FA 20 < SL 20 < Plain. This trend is different from that at 23 °C, with one of the differences being a switch in the order of FA 20 and SL 20. The amount of CH with plain cement paste was 13.79%. The increased amount of CH is a result of accelerated hydration at elevated temperature. With 20% MK, the amount of CH was 4.6%. With 20% SF, the amount of CH was 0.82%, suggesting that almost all of the CH was consumed. The amount of CH in cement paste with 20% and 40% SL was 7.98% and 3.23%, respectively. These values are much lower compared to the values at 23 ºC. 

The amounts of CH consumed by 20% and 40% SL at 23 °C were 2.92% (11.94%–9.02%) and 5.59% (11.94%–6.35%), respectively. The amounts of CH consumed by 20% and 40% SL at 50 °C were 5.81% (13.79%–7.98%) and 10.56% (13.79%–3.23%), respectively. The CH consumption almost doubled as the temperature increased from 23 °C to 50 °C, which is greater than the increase in the cumulative heat release as temperature increases. Since the amount of CH consumed is likely related to the degree of pozzolanic reaction, these results suggest that the pozzolanic reaction of SL is accelerated when curing temperature increases.

### 3.5. Compressive Strength

**Figure 10:**
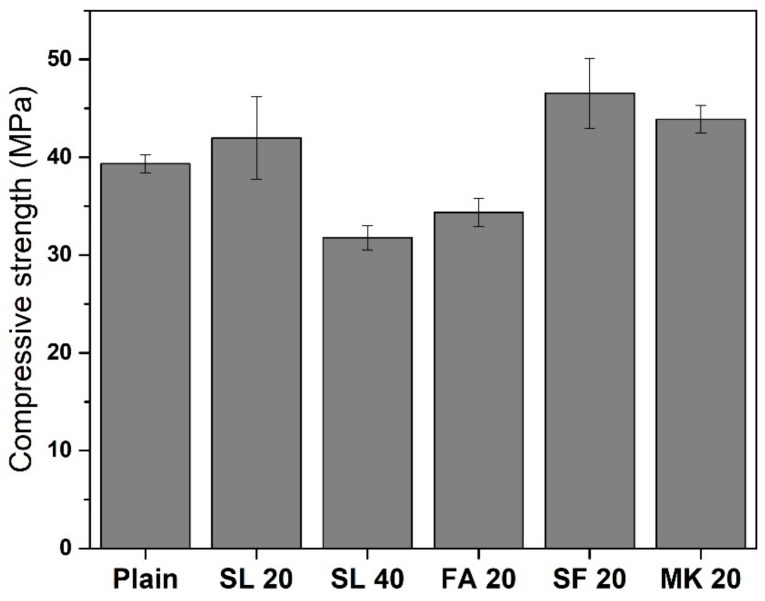
The 28-day compressive strength of concrete with various SCMs.

Figure 10 shows the 28-day compressive strength of concrete that was cured in 23 °C saturated lime solution. The mixtures, ordered in ascending order of compressive strength, are SL 40 < FA 20 < Plain < SL 20 < MK 20 < SF 20. The compressive strength of concrete with 20% SF was 46.57 MPa, which was the highest among the tested concretes. Concrete with MK also showed higher compressive strength (43.89 MPa) than that of plain concrete (39.33 MPa). Concrete with 20% Class F FA (34.37 MPa) showed smaller compressive strength than plain concrete. Concrete with 20% SL showed higher 28-day compressive strength (41.97 MPa) than that of plain concrete but considering the standard deviation of the data (4.22 MPa), the effect of SL on the 28-day compressive strength does not seem to be strongly evident.

## 4. Discussion

As mentioned in the Introduction, SL is generally thought to reduce the heat of hydration of concrete, and thus has been used for such applications. However, the results from this work, contrary to some studies [7,25,40], show that this may not be correct in truly adiabatic conditions. It is not certain if this is a result of using a different measurement system (true adiabatic conditions), or it is associated with pre-treatment of SL for activation (in this case by addition of calcium sulfate anhydrite), or for other reasons. The difference between semi-adiabatic and adiabatic response for the SL is quite clear (Figure 3 and Figure 4), and this is an important point to note. Considering the fact that current SL manufacturers typically apply various pre-treatment methods to increase the reactivity of SL, it is clear that such SL should be used with caution in mass concrete applications, since the core regions of the mass concrete may be similar to true adiabatic conditions.

Temperature rise in the core regions of mass concrete is likely to be similar to adiabatic test results (Figure 3), where SL 20 showed the highest temperature rise. On the other hand, the temperature near the surface may be located in between ambient temperature and the maximum temperature obtained by semi-adiabatic test results (Figure 4), where SL 20 showed lower temperature rise than the plain concrete. This suggests that the temperature difference between the core region and the near surface region of a mass concrete with SL 20 could be larger that of the one with plain concrete, leading to a higher possibility of surface thermal cracks. This undesired scenario may not apply to a moderately massive concrete structure since the temperature rise in the core region does not exactly follow the adiabatic condition. However, a truly massive concrete structure, such as a 5 m-thick-mat foundation, may fall into such a category. The use of slag should be very carefully considered in this case. 

Temperature and heat analyses obtained from adiabatic calorimeter and isothermal calorimetry data indicate that the concrete with 20% SL replacement had slightly higher heat of hydration than plain concrete at 23 °C and 50 °C. As the SL used in this study contains CaSO_4_, the slag may be activated at both temperatures. The CaSO_4_ increases the concentration of sulfate ions in the concrete with SL, which leads to the formation of ettringite at an early age [46,47]. As temperature increases, both the hydraulic and pozzolanic behavior of the SL seem to be enhanced. However, considering the consumption of CH by SL at 50 °C, it seems that higher temperature affects the pozzolanic reaction of SL more than the hydraulic reaction.

In order to conclusively demonstrate some of the above hypotheses, it is necessary to run a series of experiments using SL without CaSO_4_. Then, the effect of calcium sulfate on adiabatic temperature rise of concrete may be evaluated. The effect caused by the amount and type of calcium sulfate (gypsum, hemihydrate, or anhydrite) in SL as well as the effect of various pretreatment of SL will also need to be investigated. Moreover, various replacement levels of SL up to 80% should be investigated because generally 65 to 80% is considered as an optimum SL replacement range for mass concrete applications. Such experiments can confirm the findings obtained from this study.

## 5. Conclusions

According to the results shown in this work, the following conclusions can be drawn.

(1) In semi-adiabatic conditions, concrete with SL showed a reduction in the maximum temperature compared to plain concrete. However, in true adiabatic conditions, concrete with SL showed slightly higher maximum temperature than that of plain concrete. This difference is important as it may potentially lead to cracking in mass concrete structures.
(2) Cement paste with SL showed greater heat release at ambient temperature (23 °C) and elevated temperature (50 °C) compared to the cement pastes with other SCMs. Temperature enhances both hydration of the cement and pozzolanic reaction of the SCMs.
(3) The increased amount of CH consumption in cement paste with SL at the elevated temperature (50 °C) suggests that pozzolanic activity of SL is enhanced at 50 °C.
(4) The use of SL (up to 40%) may not help the reduction of the heat of hydration in a large volume of concrete (mass concrete).

## Figures and Tables

**Table 1:** Chemical compositions (mass %) and densities (g/cm^3^) of OPC and supplementary cementitious materials.

Contents	OPC	Slag	Fly Ash	Silica Fume	Metakaolin
CaO	62.29	39.92	7.94	3.43	1.46
SiO_2_	19.88	32.71	55.41	91.62	57.71
SO_3_	2.47	3.37	0.45	0.02	0.25
Al_2_O_3_	5.15	15.12	25.46	0.51	36.70
Fe_2_O_3_	3.13	0.53	8.33	0.03	2.44
MgO	3.47	6.50	1.77	0.25	0.48
K_2_O	0.91	0.58	1.41	0.37	0.65
TiO_2_	0.30	0.74	1.47	0.01	0.31
Mn_2_O_3_	0.18	0.31	0.08	0.01	-
P_2_O_5_	0.15	0.08	0.68	0.56	-
ZnO	0.09	-	0.02	-	-
Na_2_O	0.28	0.38	0.70	0.34	-
SrO	0.04	0.07	0.22	-	-
Cl	0.01	0.01	0.01	0.01	-
Density (g/cm^3^)	3.15	2.88	2.3315	2.34	2.54

**Table 2:** Mix proportions of concrete (kg/m^3^).

Type	w/b	s/a (%)	Water	Cement	Slag	Fly Ash	Silica Fume	Meta Kaolin	Fine Aggregate	Coarse Aggregate
Plain	0.35	40	205	585.71	-	-	-	-	645.60	950.13
SL 20				468.57	117.14	-	-	-	641.91	944.69
SL 40				351.43	234.29	-	-	-	638.21	939.25
FA 20				468.57	-	117.14	-	-	631.73	929.72
SF 20				468.57	-	-	117.14	-	636.14	936.20
MK 20				468.57	-	-	-	117.14	631.96	930.05

**Table 3:** The maximum temperatures and times to reach maximum temperatures of concretes tested under adiabatic and semi-adiabatic temperature conditions.

Type	Adiabatic Temperature Rise		Semi-Adiabatic Temperature Rise	
	Max Temperature (°C)	Time (h)	Max Temperature (°C)	Time (h)
Plain	84.62	41.83	63.07	19.50
SL 20	86.69	57.83	54.84	24.67
SL 40	86.98	90.67	49.83	28.33
FA 20	81.10	53.83	52.65	22.17
SF 20	79.01	54.17	53.79	23.00
MK 20	77.91	58.33	57.00	24.67
